# Report on the 2025 Indo-Pacific Health Security Alliance Meeting in Papua New Guinea: strengthening civil–military coordination for health emergency preparedness and response

**DOI:** 10.5365/wpsar.2025.16.3.1310

**Published:** 2025-08-29

**Authors:** Air Commodore Nicole dos Santos, Lieutenant Colonel (Dr) Peter Kaminiel, Sean T Casey

**Affiliations:** a Joint Health Command, Australian Defence Force, Canberra, Australian Capital Territory, Australia.; b Papua New Guinea Defence Forces, Port Moresby, Papua New Guinea.; c World Health Organization Western Pacific Regional Office, Manila, Philippines.; d School of Population Health, Faculty of Medicine and Health, University of New South Wales, Sydney, New South Wales, Australia.

The Indo-Pacific health security alliance (IPhsa) convened its most recent regional coordination meeting on 6–8 May 2025 in Port Moresby, Papua New Guinea. The meeting was hosted by the Government of Papua New Guinea and brought together over 75 participants, comprising IPhsa members, observers and technical partners – including the Pacific Community and the World Health Organization (WHO) – from 15 countries and territories across the Indo-Pacific region. (*1*) Approximately 60% of the participants were from military/security forces, while 40% represented civilian entities. Co-hosted by the Papua New Guinea Defence Force (PNGDF), the Australian Defence Force (ADF) and the United States Indo-Pacific Command (USINDOPACOM), the event focused on strengthening multisectoral and civil–military coordination for public health emergency preparedness and response. (*2*)

IPhsa was established in 2022 through a memorandum of cooperation between ADF and USINDOPACOM to counter global health security threats, strengthen regional resilience, and expand civil–military collaboration with like-minded partners and to enhance shared capabilities to safeguard the Indo-Pacific region. (*1*) The alliance provides a unique platform for civilian and military partners to share resources, undertake technical exchange and participate in simulation-based learning. IPhsa brings together partners with shared interests in advancing health security, including health authorities, military and police forces, disaster management agencies and development partners. (*1*, *3*)

The May 2025 IPhsa meeting featured plenary presentations, scenario-based table-top exercises and reflective discussions. These included a keynote presentation by the WHO Regional Office for the Western Pacific on the health-security interface in the context of the Asia Pacific Health Security Action Framework; (*4*) a presentation of the results of Papua New Guinea’s recent Civil–Military Health Security Mapping workshop; experience-sharing from participating countries, including Fiji, Indonesia, Japan, New Zealand and Singapore; and a presentation by the International Committee of the Red Cross regarding international humanitarian law. (*1*) Sessions highlighted lessons learned from recent emergencies in the region, including measles outbreaks, the COVID-19 pandemic, and the increasing frequency of concurrent and compounding natural and human-induced hazards.

The IPhsa meeting showcased several tools and frameworks to guide cross-sectoral health emergency preparedness and response. These included the *National civil-military health collaboration framework for strengthening health emergency preparedness: WHO guidance document*, the WHO *Strategic toolkit for assessing risks: a comprehensive toolkit for all-hazards health emergency risk assessment* (STAR), the WHO *Asia Pacific health security action framework*, the *International Health Regulations (2005) States Parties self-assessment annual reporting tool*, the *United Nations humanitarian civil-military coordination field handbook* (version 2.0), and others. (*2*, *4*-*7*)

Building on presentations, participants engaged in a full-day table-top emergency simulation exercise designed to test civil–military coordination, identify collaboration bottlenecks and provide a platform for future joint efforts.

Participants were asked to complete an anonymous online evaluation at the end of the meeting. In the feedback provided, participants emphasized the need for institutionalizing civil–military coordination, not only during response, but also throughout preparedness and recovery phases. Following the meeting and exercise, participants overwhelmingly indicated that they were better able to identify key actors during emergencies, that they could describe the application of key global frameworks in public health emergencies, and that they could contribute to multidisciplinary teams in health emergency response. Eighty-eight per cent of the participants reported that, because of the meeting, they had identified new opportunities for collaboration, and many said that they would advocate for the development or expansion of civil–military coordination in emergencies in the future (**Box 1**).

Box 1 **Illustrative action plans identified by meeting participants^a^**

**Table:** 

By December 2026, improve health integration and participation in relevant multisectoral regional and global civil–military forums.
By May 2026, develop a memorandum of understanding for civil–military interoperability.
Within 1 year, develop a contingency plan for disaster and health crises through collaboration with the ministry of health, security forces and other ministries/agencies, including the national disaster management office, local government, private sector, communities and academia.
Enhance civil–military relationships by developing a table-top exercise within 1 year that focuses on civil–military collaboration and partnerships.
By January 2026, establish better coordinated preparedness and response teams.
Within 1 year, strengthen the coordination of a national medical logistics support system for public health emergencies, including the development of a standard operating procedure for joint civil–military logistics and joint training of civil–military partners.
Within 1 year, review and update the memorandum of understanding between defence forces and national health authorities to strengthen civil–military collaboration.
By August 2025, set up a meeting with civilian health partners and defence stakeholders to understand the different roles and responsibilities during emergencies.
Within 1 year, establish a civil–military health committee that will include quarterly meetings, two exercises per year and joint deployments.
By 31 December 2025, organize an inter-agency technical working group for outbreak prevention, preparedness and response to achieve the 7–1-7 targets for outbreak response.^b^
Within 1 year, develop and finalize activation thresholds and response standard operating procedures for a tiered escalation model.
Within 1 year, conduct a meeting of heads of ministries to highlight and strengthen information-sharing across sectors.
By December 2025, develop a national multiagency programme to address health security and emergency response.
Within 1 year, review the existing civil-military workplan to build policies to mobilize, manage, coordinate and use resources effectively for response.

^a^ Edited for clarity and brevity.^b^ The 7–1-7 targets measure the timeliness of three outbreak milestones: detection (target of ≤ 7 days from emergence), notification (target of ≤ 1 day from detection) and completion of early response outbreak actions (target of ≤ 7 days from notification).

PNGDF’s leadership in hosting the event and in joining IPhsa as a full member underscored the country’s growing role in regional health-security efforts. Following the implementation of a hazard mapping workshop in February 2025 and a National Workshop on Advancing Civil-Military Collaboration to Strengthen Health Emergency Preparedness in Papua New Guinea only 1 week before the IPhsa meeting, Papua New Guinea’s commitment to strengthening health security action through transparent assessment and coordinated planning was evident.

As global health threats grow more complex and transboundary, initiatives such as IPhsa offer valuable fora to harmonize response efforts, build mutual understanding and create opportunities for collaboration among diverse stakeholders. Continued investment in civil–military coordination, guided by global norms and regional collaboration, will be essential to ensuring that Indo-Pacific countries remain prepared to manage future health threats quickly and effectively, protecting population health, economies, and national and regional security.
